# Hypertensive Emergency in a Spontaneous Page Kidney

**DOI:** 10.7759/cureus.41789

**Published:** 2023-07-12

**Authors:** Asha Meilstrup, Kyle Clay, Austin Burkenstock

**Affiliations:** 1 School of Medicine, University of Mississippi Medical Center, Jackson, USA; 2 Department of Medicine, University of Mississippi Medical Center, Jackson, USA; 3 Department of Medicine, G. V. (Sonny) Montgomery VA (Veteran Affairs) Medical Center, Jackson, USA

**Keywords:** resistant hypertension, spontaneous renal hematoma, page phenomenon, renal hematoma, hypertensive emergency, hypertension, page kidney

## Abstract

Page kidney or Page phenomenon is a rare cause of hypertension that results from external compression of the kidney and renin-angiotensin-aldosterone system activation. Most cases involve trauma as the precipitating factor. Page kidney can result in severe and resistant hypertension that can be challenging to treat. We present a unique case: a patient in hypertensive emergency secondary to Page kidney caused by a spontaneous subcapsular renal hematoma.

## Introduction

Page kidney is a rare and frequently missed phenomenon involving extensive activation of the renin-angiotensin-aldosterone system (RAAS) due to compression of the kidney parenchyma [1]. The compression of the intrarenal vessels simulates a low-volume state in the kidneys, activating the RAAS to increase the body’s volume status [2]. This can lead to progressively worsening systemic hypertension with high renin levels and renal ischemia. This phenomenon is often caused by a perirenal hematoma. Most of these hematomas are related to trauma, tumors, or surgery to the area [2]. For reports up to 2008, only 11% of cases of Page kidney were reported with unknown etiology [1]. This case report highlights the uncommon presentation of page kidney due to a spontaneous subcapsular renal hematoma, supported by imaging modality, and discusses its management.

## Case presentation

**Figure 1:**
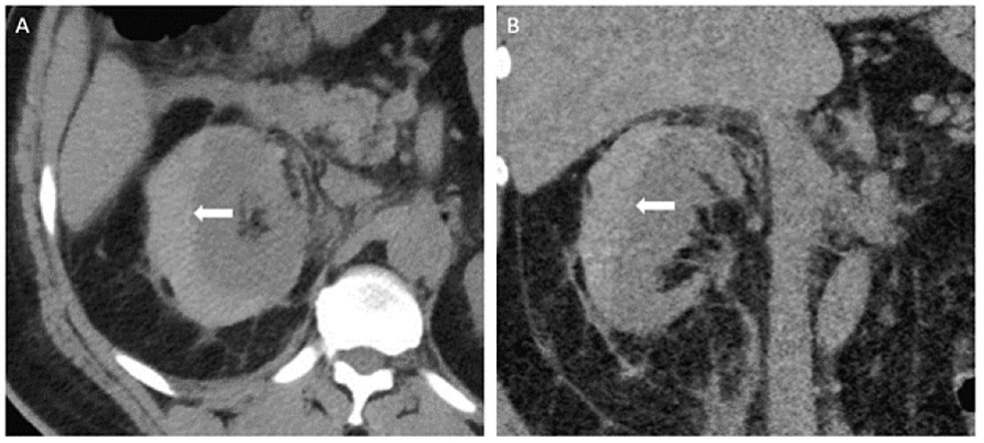
Initial imaging Axial (A) and coronal (B) non-contrasted CT scan of the abdomen/pelvis showing a subcapsular hematoma of the right kidney measuring up to 3 cm in thickness

A 53-year-old man with hypertension, hyperlipidemia, chronic kidney disease stage 3a (baseline creatinine 1.44 mg/dL), and obesity presented to the emergency department for new-onset, severe right flank pain radiating to the right groin. His home medications included amlodipine 10 mg daily and lisinopril 40 mg daily. His blood pressure was 226/142 on initial measurement. Initial creatinine was 1.66 mg/dL. A computed tomography (CT) scan of his abdomen/pelvis revealed a subcapsular hematoma of the right kidney measuring up to 3 cm in thickness (Figure 1). The combination of imaging findings and hypertension was concerning for Page kidney. He had no prior imaging and denied any recent trauma or bleeding disorders. He was placed on a nicardipine infusion and transferred to the medical intensive care unit. His pain was controlled with a multi-modal pain regimen, including large doses of intravenous hydromorphone. His hemoglobin remained stable at around 12.0 g/dL and his creatinine peaked at 2.5 mg/dL.

**Figure 2:**
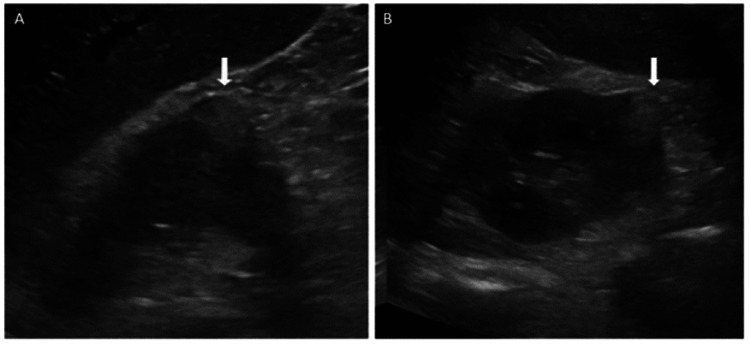
Follow-up imaging Renal ultrasound (A & B) of the right kidney showing a 2.5 x 2 x 2.2 cm oval density representing a resolving hematoma

After a multidisciplinary discussion with urology, nephrology, and interventional radiology, the decision was made not to intervene and to monitor the hematoma. His anti-hypertensive regimen was titrated until he was ultimately controlled on hydralazine 100 mg three times daily, isosorbide mononitrate 90 mg daily, amlodipine 10 mg daily, carvedilol 6.25 mg twice daily, and lisinopril 10 mg daily. Repeat CT abdomen/pelvis prior to discharge did not show any increase in the size of the hematoma. Once his pain and hypertension were stabilized, he was sent home with remote blood pressure monitoring and close follow-up. His creatinine was improved at 2.16 mg/dL on the day of discharge. At follow-up six months later, the size of the hematoma was decreased on renal ultrasound (Figure 2). His blood pressure remained well-controlled on the same anti-hypertensive regimen and has yet to decrease while outpatient. His creatinine had further improved to 1.43 mg/dL, which is likely his new baseline.

## Discussion

Knowledge of Page kidney was first described by Irvine Page in 1939 when he experimented with animal kidneys by wrapping them in cellophane [1]. He observed that the animals developed hypertension after four to five weeks. This hypertension resolved after a nephrectomy, indicating that the kidney was responsible. The first diagnosis of Page kidney was then described by Page and Engle in the 1950s in a young man with a subcapsular renal hematoma due to a football injury [1]. From 1955-2008, roughly 108 cases of Page kidney were described in the literature [1]. Initially, the most likely group of individuals diagnosed with Page kidney were young, healthy male athletes who had undergone physical trauma to the kidneys during sporting events. The average age of diagnosis is now 38 years old, and many other etiologies have been described including renal biopsies, renal cysts/tumors, transplants, or non-sports-related trauma [1].

Patients with Page kidney often do not have any clinical symptoms other than resistant hypertension with average blood pressure at the time of diagnosis of 177/95 [1]. Some symptoms of end-organ damage related to hypertension may include chest pain or dyspnea from acute coronary syndrome or heart failure, weakness/dizziness/altered mental status from cerebrovascular accident/hemorrhage, or vision changes from retinal hemorrhages or papilledema. In young patients with hypertension and known abdominal trauma or renal tumors, Page kidney should be included on the differential. It is important to note that the onset of hypertension from a trauma-induced Page kidney may present years after the causal event, so a thorough history must be obtained. For this reason and the lack of unique clinical presentation, Page kidney must be considered in the differential diagnosis for secondary hypertension [3].

Renal ultrasound is often the imaging modality of choice in diagnosing Page kidney [2]. A CT scan is also beneficial if there is a high suspicion of Page kidney or a tumor that may be too small for an ultrasound to detect and is often completed after initial ultrasound imaging [1]. Elevated renin is another significant finding for Page kidney, but many other diagnoses may also cause elevated renin (renin-producing tumors, adrenal insufficiency, etc.), making this test less specific.

Treatment for Page kidney varies and is dependent on the cause. It is essential to rule out a tumor as the cause of Page kidney, as this would lead down a specific treatment path separate from hematoma-induced Page kidney [4]. If ultimately determined to be the latter, providers must assess for causes of the bleeding, such as recent surgery, anti-coagulants, bleeding disorders, arteriovenous malformations, or vasculitis. Rarely, an etiology for Page kidney goes undiscovered by clinical history or workup, but imminent treatment is still required. The mainstay treatment of hematoma-induced Page kidney was originally surgical drainage of the hematoma or complete nephrectomy. We currently do not have a standard treatment for Page kidney and opt to treat it on a patient-by-patient basis [2]. First-line treatments often include anti-hypertensives to control blood pressure as the hematoma resorbs. Since the resorption of the hematoma decreases the compression on the kidney, this leads to decreased RAAS activation. Close follow-up and de-escalation of anti-hypertensives is essential. Surgical intervention may be required if the patient is hemodynamically unstable or if a hypertensive emergency with worsening end-organ damage continues to ensue.

Our patient presented in a hypertensive emergency. His uncontrolled hypertension may have contributed to the severity of his presentation, but his history presented no significant risk factors for Page kidney. The CT scan revealed a 3 cm subcapsular renal hematoma, and when clinically correlated with his degree of hypertension, was concerning for Page kidney. After a multi-disciplinary discussion with urology, nephrology, and interventional radiology, the decision was made to monitor the hematoma carefully. It was determined that the hematoma was likely tamponading any additional bleeding. Therefore, the concern in pursuing percutaneous drainage was that it could lead to worsening blood loss and possible hemodynamic instability. Medical management proved to be the safest option unless his condition greatly declined. This was done by closely monitoring his flank pain to ensure it was not worsening from baseline, ensuring that his hemoglobin level was stable and that his blood pressure was titrated to safe levels. Intravenous contrast was not used for the CT scans in the rare case that it worsened his renal function. His blood pressure regimen included hydralazine 100 mg three times daily, isosorbide mononitrate 90 mg daily, amlodipine 10 mg daily, and carvedilol 6.25 mg twice daily. Of note, his home lisinopril was held on admission to avoid further worsening of his renal function and restarted before discharge. His renal function improved as his blood pressure improved, and the hematoma size remained stable (based on serial CT scans).

Our patient’s hypertension was resistant and challenging to control. No tumor or other cause was identified on repeat imaging, but he will still need a CT scan with contrast in the future to identify any small, missed tumor. However, the resolving hematoma and lack of overt mass are reassuring. Once our patient was stable, he was discharged with close follow-up with nephrology, urology, and primary care. Our patient was instructed to monitor his blood pressure daily and work closely with his primary care provider to de-escalate his anti-hypertensive medications as the hematoma reabsorbed and blood pressure improved. Upon follow-up approximately six months later, the hematoma decreased on imaging, and his hypertension was controlled on his oral medications. This case was successfully managed medically, and our patient’s renal function returned to baseline. Page kidney is not often on the differential diagnosis for secondary causes of hypertension, but as this case demonstrates, early diagnosis and treatment are imperative [5].

## Conclusions

Page kidney is a rare but severe illness that can lead to prolonged and uncontrolled hypertension, a hypertensive emergency, and significant renal damage. Treatment includes detecting the source of kidney compression, extensive use of anti-hypertensives, and occasionally requiring surgical intervention. For these reasons, Page kidney should be included more frequently in the differential diagnoses for patients with hypertension. Spontaneous Page kidney is a rare phenomenon and was an unexpected finding when this patient presented. We hope to raise awareness for future clinicians when a patient presents with flank pain and a hypertensive emergency.
